# Microacoustic Metagratings at Ultra‐High Frequencies Fabricated by Two‐Photon Lithography

**DOI:** 10.1002/advs.202200990

**Published:** 2022-04-24

**Authors:** Anton Melnikov, Sören Köble, Severin Schweiger, Yan Kei Chiang, Steffen Marburg, David A. Powell

**Affiliations:** ^1^ Monolithically Integrated Actuator and Sensor Systems Fraunhofer Institute for Photonic Microsystems IPMS 01109 Dresden Germany; ^2^ School of Engineering and Information Technology University of New South Wales Canberra 2612 Australia; ^3^ Chair of Vibro‐Acoustics of Vehicles and Machines Technical University of Munich 85748 Garching Germany

**Keywords:** acoustics, metamaterials, refraction, thermoviscous effects, two‐photon polymerization, ultrasound

## Abstract

The recently proposed bianisotropic acoustic metagratings offer promising opportunities for passive acoustic wavefront manipulation, which is of particular interest in flat acoustic lenses and ultrasound imaging at ultra‐high frequency ultrasound. Despite this fact, acoustic metagratings have never been scaled to MHz frequencies that are common in ultrasound imaging. One of the greatest challenges is the production of complex microscopic structures. Owing to two‐photon polymerization, a novel fabrication technique from the view of acoustic metamaterials, it is now possible to precisely manufacture sub‐wavelength structures in this frequency range. However, shrinking in size poses another challenge; the increasing thermoviscous effects lead to a drop in efficiency and a frequency downshift of the transmission peak and must therefore be taken into account in the design. In this work three microacoustic metagrating designs refracting a normally incident wave toward −35° at 2 MHz are proposed. In order to develop meta‐atoms insensitive to thermoviscous effects shape optimization techniques incorporating the linearized Navier–Stokes equations discretized with finite element method are used. The authors report for the first time microscopic acoustic metamaterials manufactured using two‐photon polymerization and, subsequently, experimentally verify their effectiveness using an optical microphone as a detector in a range from 1.8 to 2.2 MHz.

## Graphical abstract

This work presents microacoustic metagratings for passive wavefront manipulation of ultra‐high frequency ultrasound. The designs are created with topology and shape optimization techniques by incorporating the thermoviscous effects and manufactured with two‐photon lithography. The successful experimental demonstration of the effectiveness of the designs opens a new route for acoustic metamaterials in ultrasonic applications with high spatial resolution.

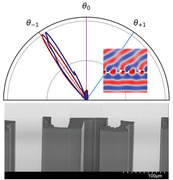

## 1 Introduction

Acoustic metamaterials make it possible to design unique material properties that do not occur in nature,^[^ ^1^, ^2^, ^3^, ^4^ ^]^ for example negative bulk modulus and negative dynamic mass density.^[^ ^5^, ^6^ ^]^ Because of these unusual properties, acoustic metamaterials have drawn attention in the context of unconventional acoustic wave manipulation. This opened up a wide range of applications, such as acoustic cloaking,^[^ ^7^, ^8^, ^9^ ^]^ acoustic barriers of subwavelength thickness,^[^ ^10^, ^11^, ^12^ ^]^ flat acoustic lenses,^[^ ^13^, ^14^, ^15^ ^]^ and ultrasonic imaging with subwavelength resolution.^[^ ^16^, ^17^, ^18^ ^]^ In the case of the latter two examples, the class of gradient metasurfaces including reflecting^[^ ^19^, ^20^ ^]^ and refracting^[^ ^21^, ^22^, ^23^, ^24^, ^25^, ^26^, ^27^ ^]^ designs were proven by experiments to be an effective passive approach for wavefront control, for example, for acoustic Fresnel lenses.^[^ ^19^, ^22^, ^24^, ^25^, ^27^, ^28^ ^]^ However, the wavelength limits the resolution and therefore for high resolution applications the frequency rises to high or even ultra‐high frequency ultrasound requiring scaling of the metasurface geometries. When speaking of airborne ultrasound, the ultra‐high frequency range starts from 0.5 MHz, where thermoviscous layers at sound‐hard boundaries introduce stronger losses.^[^ ^29^, ^30^ ^]^ Because gradient acoustic metasurfaces use very thin acoustic channels and are often enhanced by Helmholtz resonators, the thermoviscous effects can become severe enough to interfere with the proper functioning of the design. Bianisotropic metagratings^[^ ^31^, ^32^, ^33^, ^34^, ^35^, ^36^ ^]^ are promising for this ultra‐high frequency range, since their geometrical features are close to the wavelength and, therefore, much larger than in the case of gradient metasurfaces. However, such scaling has never been investigated due to challenges in manufacturing such microscopic structures and the need to make the design insensitive to thermoviscous losses. Due to the latest developments in the additive manufacturing of microscopic structures, in particular two‐photon lithography, one of the biggest hurdles on the way to the experimental realization of microacoustic metagratings can now be overcome.

**Figure 1:**
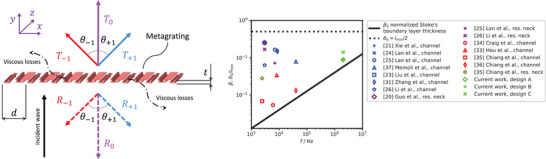
Wavefront directions generated by microacoustic metagrating and design aspects. a) Microacoustic metagrating with three diffraction orders and viscous losses with a normal incident wave. Refracted and reflected wavefronts are indicated by solid and dashed arrows, respectively. The diffraction order is indicated by the subscripts. The global metagrating parameters are the lattice constant *d* and the grating thickness *t*. b) Normalized Stoke's boundary layer thickness *β*_S_ and Stoke's boundary layer *δ*_S_ compared to the thinnest channel dimension *l*_min_ for a selection of acoustic metamaterial structures taken from the literature.^[^ ^20^, ^21^, ^23^, ^24^, ^25^, ^26^, ^27^, ^34^, ^35^, ^36^, ^37^ ^]^

Acoustic metagratings^[^ ^38^ ^]^ are periodic arrays of discrete meta‐atoms, which can diffract the incident acoustic energy via multiple diffraction orders as shown in **Figure** **1**a. The number of modes *n* and associated diffraction angles *θ*_*n*_ are determined by the acoustic wavelength λ and the metagrating lattice constant *d*, based on Bragg's condition, that is, *d* = *n*λ/sin *θ*_*n*_. The energy redirection to different diffraction orders is attributed to the scattering properties of the meta‐atoms and is determined by their geometric shape. It has recently been shown that by including an additional degree of freedom, represented by the Willis coupling parameter, the acoustic analogy of bianisotropy in electrodynamics, more efficient metamaterial designs can be created.^[^ ^39^, ^40^, ^41^, ^42^, ^43^ ^]^ A non‐zero Willis coupling parameter is linked to geometric asymmetry of the meta‐atom^[^ ^42^, ^43^ ^]^ and in case of metagratings it decouples the +*n*th and −*n*th diffraction orders from each other. Based on this mechanism, acoustic metagratings have been experimentally demonstrated in the audible frequency range; at 6 kHz by Craig et al.,^[^ ^34^ ^]^ at 8.2 kHz by Hou et al.,^[^ ^33^ ^]^ and in the range from 2.43 to 2.54 kHz by Chiang et al.^[^ ^35^ ^]^ Due to coarse geometric features compared to the wavelength, acoustic metagrating are scalable to low‐frequency ultrasound range, as demonstrated by Chiang et al.^[^ ^36^ ^]^ with a 40 kHz design.

Microacoustic metagratings consist of micro‐fabricated structures, the natural consequence of this microscale being the significant influence of thermoviscous effects. The viscosity begins to strongly contribute when the Stokes's boundary layer thickness $\delta_{S}=2\pi \sqrt{2\mu /(\omega \rho_{0})}$ being related to the well known viscous boundary layer $\delta_{v}=\sqrt{\mu /(\omega \rho_{0})}$ as $\delta_{S}=2\pi \sqrt{2}\delta_{v}$ approaches the geometrical dimensions of the channels.^[^ ^29^, ^30^ ^]^ Here μ is dynamic viscosity, *ρ*_0_ is equilibrium density, and *ω* = 2*π* *f* is angular frequency. In metagratings, the dimensions of the meta‐atoms are close to the wavelength *λ* = *c*_0_/*f*, which, however, decreases faster with increasing frequency *f* than the thickness of the boundary layers (*c*_0_ is speed of sound). Therefore we introduce a normalized Stoke's boundary layer thickness

(1) $$\beta_{S}=\frac{\delta_{S}}{\lambda }=\frac{\sqrt{f}}{c_{0}}\sqrt{\frac{4\pi \mu }{\rho_{0}}}$$

that relates the boundary layer thickness to the wavelength and then indirectly to the metagrating dimensions. Accordingly, the thermal boundary layer $\delta_{t}=\sqrt{k/(2\pi f\rho_{0}C_{p})}$ being linked to *δ*_v_ by the Prandtl number Pr as $\delta_{t}=\delta_{v}/\sqrt{\mathrm{Pr}}$ ^[^ ^29^, ^30^ ^]^ can be normalized as

(2) $$\beta_{t}=\frac{\sqrt{f}}{c_{0}}\sqrt{\frac{k}{2\pi \rho_{0}C_{p}}}$$

with *k* as thermal conductivity and *C*_*p*_ as heat capacity at constant pressure. Figure 1b illustrates the normalized Stoke's boundary layers *β*_S_ over the frequency, where it can be observed that a change of two order of magnitude in frequency (scaling from audio range to ultra‐high frequency ultrasound) leads to an order of magnitude change in boundary layer thickness.

To demonstrate the current state of the art, Figure 1b plots the gradient metasurfaces and metagratings reported in the literature, showing the relation of the thinnest channel dimension *l*_min_ to *δ*_S_. We note that previously reported metamaterial structures from the literature in Figure 1b are limited by 40 kHz. In the case of gradient metasurfaces (Figure 1b, blue markers), the ratio *δ*_S_/*l*_min_ ranges from 6% to 25%, where thermoviscous effects can be expected. If however, these gradient metasurface designs would be scaled to ultra‐high frequency ultrasound (shifting in parallel to the *β*_S_ curve), in most cases the Stoke's boundary thickness would exceed the channel width (Figure 1b, black dotted line) significantly changing the designed phase and leading to high absorption. Metagratings offer a huge potential to reduce losses by using a channel width close to the wavelength (Figure 1b, red markers). Nevertheless, scaling to ultra‐high frequency ultrasound is still a challenge, since the Stoke's boundary thickness is close to 10% of the channel width, where thermoviscous effects need to be taken into account in the design process.

Here we report for the first time on the largely unexplored concept of microacoustic metagrating in airborne ultra‐high frequency ultrasound, as a route to use acoustic metamaterials for flat acoustics, ultrasound imaging, and acoustic spectroscopy with high spatial and temporal resolution, where it is necessary to operate at ultra‐high frequency ultrasound. The target frequency in this work is set to 2 MHz, which is far above the frequency range previously discussed in literature (see Figure 1b). We choose *λ* and *d* in a range in which only three propagating diffraction orders (−1, 0, and +1) are supported, for both refracted and reflected waves. With a view to demonstration of a diffraction angle that allows a sufficiently short focal length for flat acoustic lenses, for example, the target angle is set to *θ*_−1_ = −35.0°, while the wave is incident at 0°. The resulting lattice constant of the metagrating *d* = 299 µm is given by the Bragg's condition, such that the −1st diffraction order is aligned with the desired angle. As a result, there are six different directions in which the wave can be redirected by the metagrating, three transmitted (*T*_−1_, *T*_0_, and *T*_+1_) and three reflected (*R*_−1_, *R*_0_, and *R*_+1_) diffraction orders. Because of *β*_S_ ≈ 10^−1^, we expect considerable viscous losses that open an additional (seventh) energy channel, namely absorption *α*. In order to design meta‐atoms for microacoustic metagratings, we apply the linearized Navier–Stokes equations implemented within the finite element method (FEM) framework. We use different design strategies, all of which are finalized through FEM‐based shape optimization including thermoviscous effects, maximizing the energy transmitted into the −1st diffraction order. Finally, we design and manufacture three different microacoustic metagratings using two‐photon polymerization. This manufacturing process has not yet been explored for acoustic metamaterials and we demonstrate for the first time its application to acoustic metagratings. Unidirectional acoustic beams achieved by our advanced designs are attributed to the bianisotropic properties of the optimized meta‐atoms and to the improvement of the models compared to the commonly used lossless approaches. Although part of the acoustic energy is dissipated due to the unavoidable effect of viscous losses in the narrow slits, our numerical and experimental results demonstrate that highly directional refraction can be achieved. The metagrating structures presented can be used for most ultrasound applications, for example flat acoustic lenses for ultrasound imaging with a high temporal and spatial resolution or the directional shaping of high‐frequency ultrasonic transducers for gesture recognition.

## 2 Results

### 2.1 Can Thermoviscous Effects be Neglected?

**Figure 2:**
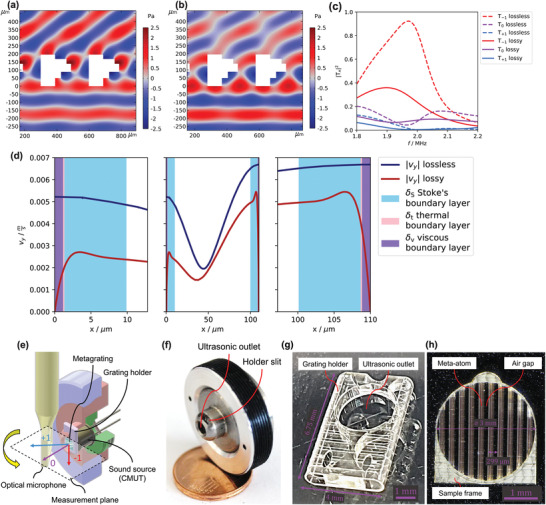
Lossless metagrating design and experimental setup a) Real part of the pressure at the peak frequency in lossless case. b) Real part of the pressure at the target frequency 2 MHz under consideration of thermoviscous effects. The transmitted amplitude is reduced and the interference with other diffraction orders can be observed. c) Transmission coefficients for the lossless model and the model with thermoviscous effects. d) Comparison of the *y* velocity *v*_*y*_ at 2 MHz in the narrowest slit between two meta‐atoms of design A. The right and left panels show a detailed view of the curves close to the boundary. e) The rotatable holder including transducer and the moving laser microphone. f) Photograph of the rotatable holder. g) Micrograph of the grating holder created by two‐photon lithography. h) Micrograph of the microacoustic metagrating sample.

The first metagrating (design A) is designed based on the Helmholtz equation, which does not consider the viscosity of air and is evolved from a 2D model assuming an infinite number of meta‐atoms. For the optimization of lossless structures, simple geometrical shapes are used in combination with a semianalytical model based on the superposition of waveguide modes similar to ref. [36]. The final shape of the meta‐atom consists of five rectangles, which add together to a thickness *t* = 196 µm, see **Figure** **2**a. This design promises |*T*|^2^ ≈ 0.95 around 2 MHz when no losses are considered, where the numerical results are shown in Figure 2c as dashed line with |*T*|^2^ in red (*T* stands for complex transmission coefficient). However, when the more accurate linearized Navier–Stokes equations are used, transmission toward −1st diffraction order is strongly reduced (see Figure 2b), while the peak shifts to 1.90 MHz with only |*T*_−1_|^2^ = 0.36 in Figure 2c (red solid line). Furthermore, we observe a redistribution of the transmitted energy to the other diffraction orders and not just a reduction of all magnitudes due to losses as might be expected. The transmission toward 0th order is even bigger as in the lossless case between 1.94 and 2.00 MHz, which means that the design requires further optimization.

In order to review the differences between both modeling strategies, the harmonic fluid velocity in *y*‐direction at the narrowest location between two meta‐atoms is shown in Figure 2d. In the lossy case (red curves) the velocity is reduced compared to the lossless case (blue curves), while it is zero at the boundary. Furthermore the entire profile deviates from the lossless case, especially within the Stoke's boundary layer (light blue region). It should be noted, that the assumption of the zero velocity at the boundary is only valid for Knudsen number Kn<0.01, which means a channel width <7 µm. In contrast to the viscous effects, the observed shift of the |*T*_−1_|^2^ peak toward lower frequencies could be linked to the thickness of the thermal boundary layer *δ*_t_, despite being only around 1% of the channel width. Similar effects are known from narrow slits^[^ ^29^ ^]^ and from resonance based bianisotropic acoustic meta‐atoms.^[^ ^42^, ^43^ ^]^ However, it is still unclear whether the reduction in transmission and the shift in the peak frequency can be resolved experimentally.

To characterize the designed and fabricated microacoustic metagratings we use the experimental setup shown in Figure 2e combining a grating mounting with an ultra‐high frequency capacitive micromachined ultrasonic transducer with a center frequency of 2 MHz (see Supporting Information), a laser microphone and precise motorized stages.^[^ ^44^ ^]^ For experimental realization, a finite number of 9 meta‐atoms is extruded into the third dimension *z* and placed at the end of the ultrasonic aperture with a diameter of 3 mm. While the transducer emits a plane wave toward the metagrating through the ultrasound aperture, the microphone detects the transmitted waves as pressure oscillations at different angles and radii in the *xy*‐plane located in the middle of the metagrating. The mounting includes a rotatable holder (Figure 2f), the grating holder (Figure 2g), and the metagrating sample (Figure 2h). The metagrating samples are manufactured by two‐photon polymerization, while the mechanical properties of the resulting polymer IP‐S are sufficient to create an impedance contrast close to a hard boundary for airborne sound.

**Figure 3:**
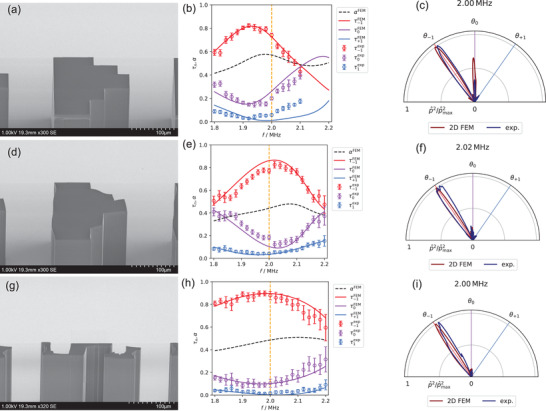
Designed and manufactured microacoutic metagratings and experimental results. a–c) Design A, semi‐analytically optimized lossless metagrating. Design B, reoptimized metagrating including thermoviscous effects. g–i) Design C, broadband and subwavelength microacoustic metagrating. (a,d,g) SEM micrograph of the meta‐atom manufactured by two‐photon lithography. (b,e,h) Experimentally and numerically determined normalized transmission *τ*_*n*_ and numerically determined absorption *α*. (c,f,i) Directivity pattern around the target frequency, determined with a finite 2D FEM model (dark‐red line) and measured (dark‐blue line, $\hat{p}$ stands for pressure amplitude).

**Figure** **3**a shows a scanning electron microscope (SEM) image of the cross section of the microscopic metagrating manufactured by two‐photon lithography. We define the normalized transmission

(3) $$\tau_{n}=\frac{|T_{n}{|}^{2}}{\sum_{m}{\left|T_{m}\right|}^{2}},\;m\in \left\{0,\pm 1\right\}$$

to characterization the performance of the metagrating. The measured and numerically calculated normalized transmission is shown in Figure 3b, while the simulations of the unnormalized transmission are included in the Supporting Information. Here we observe how accurately the experimental results match the simulation with thermoviscous effects. The numerically determined maximum *τ*_−1_ peak is located at 1.93 MHz with *τ*_−1_ = 0.82, while in the experiment we observe the peak at 1.92 MHz with *τ*_−1_ = 0.82 ± 0.02. In addition, the numerically predicted crossing point of *τ*_−1_ and *τ*_0_ around 2.10 MHz is also present in the experiment. From the simulation, we observe that half of the energy is absorbed in the metagrating around the target frequency (see Figure 3c, black dashed line), while the maximum absorption is located at 1.98 MHz with *α* = 0.58. The simulated and measured directivity profile at the target frequency 2 MHz is shown in Figure 3c, where we see strong refraction toward the intended angle *θ*_−1_ = −35°. One option to increase the performance is to use the peak frequency at 1.92 MHz, which is 4% below the target frequency, but this would change the diffraction angle to *θ*_−1_ ≈ −37°. However, this is an unacceptable compromise for flat acoustic lenses consisting of several different metagrating areas, for example, since it would lead to defocusing. Therefore, we conclude that thermoviscous effects must not be neglected during the design of microacoustic metagratings.

### 2.2 Reoptimization Including Thermoviscous Effects (Design B)

To overcome the drop in efficiency and frequency downshift of the peak, the design is shape reoptimized using FEM including thermoviscous losses based on linearized Navier–Stokes equations. During the optimization the free shape boundary method^[^ ^45^ ^]^ is used, while the shape deformation was limited to *λ*/4 from initial boundary location. The lossless design is used as the initial geometry, while the metagrating thickness *t* is fixed. The optimization target is set to maximize the transmission toward −1st diffraction order and the maximum value achieved is |*T*_−1_|^2^ = 0.45. The final geometry of design B manufactured using two‐photon polymerization and is shown in Figure 3d. It can be seen that the optimal structure resembles the initial design A and most of the geometric features are maintained.

The comparison between numerically and experimentally determined normalized transmission is shown in Figure 3e. The experimental result matches the numerics with high accuracy considering the peak and the crossings. From the numerics we observe the peak at 2.02 MHz with *τ*_−1_ = 0.87. This is confirmed by the experimental result with *τ*_−1_ = 0.83 ± 0.03 at 2.02 MHz. In addition, in FEM, as well as in the experiment *τ*_−1_ and *τ*_0_ come very close to each other around 1.80 and 2.20 MHz. Compared to initial design A, the model suggests that the absorption coefficient *α* is reduced in the investigated frequency range, while it is particularly observable around 2 MHz, where the absorption coefficient *α* drops from 0.57 to 0.44. At the target frequency there is a small performance drop (see Figure 3e), which cannot be explained by the numerical model, but makes operation at the target frequency slightly less attractive as at the neighboring frequency steps. Subsequently, viewing the measured directivity pattern at the target frequency would give a result similar as for design A. Therefore, Figure 3f shows the simulated and measured directivity at 2.02 MHz (1% above the target, *θ*_−1_ ≈ −34.6°) with *τ*_−1_ = 0.83 being an improvement compared to design A with *τ*_−1_ = 0.82 at 4% lower frequency.

### 2.3 Broadband and Subwavelength Microacoustic Metagrating (Design C)

To tackle the demand for broadband and subwavelength metagratings, we introduce a new design C, which is the result of a combination of topology optimization with shape optimization. Furthermore, thinner metagratings in general have shorter channel length, which can help to reduce unwanted absorption. In the first step a new geometry is created by topology optimization aiming for maximum *T*_−1_ without the consideration of thermoviscous losses. The optimization domain is a wall of infinite length and a thickness *t* = *λ*/4, which subsequently is the thickness limit in that step. In the second step, the newly created geometry is taken as the initial geometry for a shape optimization under consideration of thermoviscous losses. Again the free shape boundary method^[^ ^45^ ^]^ with deformation limitation of *λ*/4 is used. The final geometry promises |*T*_−1_|^2^ = 0.41 at the target frequency with a thickness of only *t* = 50 µm ≈ 0.29*λ*. It should be noted that the resulting meta‐atom consists of two separate bodies, which is surprising since it was not specified. To the best of our knowledge, such a configuration is new and unexplored in the context of acoustic metagratings.

Figure 3g shows an SEM image of the manufactured metagrating. The shape includes thin walls and jagged edges, which are accurately recreated by two‐photon lithography. The experimentally and numerically determined transmission are shown in Figure 3h, where we observe quite high amount of energy in the −1 diffraction order. From the numerics the *τ*_−1_ peak is expected at 1.98 MHz with *τ*_−1_ = 0.90, while in the experiment the peak is located at 1.96 MHz with *τ*_−1_ = 0.90 ± 0.02. In addition, *τ*_−1_ = 0.88 ± 0.04 at the target frequency, being better than the peak values of previous designs A and B. Furthermore, we note that in the frequency range between 1.80 and 2.08 MHz *τ*_−1_ > 0.80, which is broadband compared to previous results. The absorption (see Figure 3h, black dashed line) with *α* = 0.49 at the target frequency is slightly higher than in the design B (see Figure 3e), but still much lower than in the design A. Taking a look at the simulated and measured directivity pattern in Figure 3i reveals strong diffraction toward −1st order with other orders being negligible. Although this new design has thinner channels with dimensions closer to the Stoke's boundary layer thickness δ_S_ (see Figure 1b, green cross), it demonstrates the best experimental performance from the investigated geometries.

## 3 Discussion

How could the unexpected better performance of the subwavelegth design C be explained, when the range of the possible shapes is significantly limited due to the bounded thickness? This is due to a different topology of the meta‐atom consisting of two separate bodies, which was found through the naivety of the topology optimization. We can gain further insight by looking at this from the perspective of the fluidic channels, where, contrary to all previously reported acoustic metagrating designs, here we have two channels instead of a single one within a period. The dimensions of these channels are different, resulting in a transmission phase shift between the channels that is comparable to gradient metasurfaces. We note that for a gradient metasurface, two channels is the absolute minimum to break symmetry, however typically more are required to match the required phase distribution. Bianisotropic meta‐atoms consisting of multiple separated bodies have been already discussed in the optics^[^ ^46^ ^]^ and in the acoustics, although limited to multiple solid cylinders^[^ ^47^, ^48^ ^]^ or to a cylindrical shape with a resonant cavity.^[^ ^42^, ^43^ ^]^

In the current work we have demonstrated the microacoustic metagratings in air, but the aforementioned applications are at least as important for waterborne ultrasound. Using the normalized expressions for boundary layer thicknesses in Equations (1) and (2) our results can be transferred to other media, particularly water. Considering water with dynamic viscosity μ^water^ = 1.0093 × 10^−3^ Pa s, speed of sound *c* ^water^ = 1418.1 m s^−1^, and equilibrium density $\rho_{0}^{\mathrm{water}}=998.2\;\text{kg}\;m^{-3}$ the current airborne study at 2 MHz is similar to a waterborne study at 559 MHz with a grating constant of *d* = 4.63 µm from the view of viscosity. However, the normalized thermal boundary layer of thickness *β*_t_ has also non‐negligible effects, leading to a second similarity parameter *β*_t_ linked by the Prandtl number to *β*_S_. Since the Prandtl number of water Pr^water^ = 7.1 (thermal conductivity *k* ^water^ = 0.59423 W m^−1^ K^−1^ and heat capacity at constant pressure $C_{p}^{\mathrm{water}}=4186.9\;J\;{\text{kg}}^{-1}\;K^{-1}$) is one order of magnitude larger than that of air Pr^air^ = 0.71, the normalized thermal boundary layer *β*_t_ is smaller and, from the point of view of *β*_t_, the present study is comparable to 5.6 GHz with *d* = 461 nm in water. Even if thermal and viscous effects in our numerical model cannot be separated, the viscosity is the dominant source of the losses and *β*_S_ is the more relevant similarity parameter. From this we conclude that our results can be interpreted to expect a feasibility of microacoustic metagratings in water above 500 MHz, which are considered to be deep ultra‐high frequency for waterborne ultrasound. This only applies if the metagrating material produced has an impedance contrast comparable to the sound‐hard boundary, which is not the case when using the same polymers as in the current work. This can be solved by manufacturing glasses with two‐photon lithography,^[^ ^49^ ^]^ otherwise the structural dynamics must be included in the design process via the fluid‐structure interface.

The focus of this manuscript is at −1st transmission order with a constant lattice constant *d*. In order to apply the approach presented here for the design of an acoustic lens, it is necessary to develop microacoustic metagrating cells with different *d* and additionally to control different diffraction orders, for example, −2, −1, 0, +1, +2, etc. Reference 36] demonstrates such an approach in reflection, where the same semi‐analytical method as for design A was used to generate different metagrating cells. As an outlook, the optimization approach presented in our work can be combined with ref. [36] to develop acoustic lenses for transmission at ultra‐high frequencies. Furthermore, the potential of fabricating metamaterials directly onto MEMS devices using two‐photon lithography^[^ ^50^ ^]^ offers additional advances in the integration of acoustic metamaterials into MEMS.

## 4 Conclusions

We have demonstrated the feasibility of microacoustic metagratings fabricated by two‐photon polymerization for control of ultra‐high frequency ultrasound. We proved experimentally that anomalous refraction at ultra‐high frequencies in air (⩾2 MHz) is possible, which is of particular interest for flat acoustics and ultrasonic imaging with high spatial and temporal resolution. We have shown numerically and experimentally that the unavoidable thermoviscous effects within metagratings result in efficiency drop and frequency downshift of the peak, when the designs are based on the commonly used lossless Helmholtz equation. This problem is mainly caused by the viscous and thermal boundary layer thicknesses being close to the geometric dimensions of the meta‐atoms when shrinking to the microscopic dimensions required for ultra‐high frequencies. Therefore thermoviscous effects should be explicitly modeled to optimize the metagrating designs to overcome the drop in performance and frequency downshift. These results push forward research and innovation in the field of ultra‐high ultrasound waves in air and other viscous fluids.

## 5 Experimental Section

### Numerical Model

For numerical solution the linearized Navier–Stokes model was solved using the COMSOL Multiphysics software. An infinite metagrating in 2D space was assumed and therefore only a single strip of the length *d* was modeled. To ensure the periodicity of the solution a Bloch–Floquet boundary condition was applied. The infinite domain extension was modeled by using perfectly matched layers (PML) above and below the metagrating.

In order to obtain plane wave expansion coefficients a surface *A* of the size *d* × *d* was defined before (reflection, *R*) and after (transmission, *T*) the metagrating leading to

(4) $$\begin{array}{cc} a_{n}^{T} & =\frac{1}{A_{T}}\int_{A_{T}}p\left(r,f\right)e^{ik_{n}^{T}r}dA_{T}\\ a_{n}^{R} & =\frac{1}{A_{R}}\int_{A_{R}}p\left(r,f\right)e^{ik_{n}^{R}r}dA_{R}\\ a_{\mathrm{inc}} & =\frac{1}{A_{R}}\int_{A_{R}}p\left(r,f\right)e^{ik_{\mathrm{inc}}r}dA_{R} \end{array}$$

with *n* ∈ {0, ±1} being the diffraction order, *p*(**r**, *f*) being the complex valued pressure, and **r** being the location vector. The wave vector **k** is defined as

(5) $$\begin{array}{cc} k_{n}^{T} & =e_{y}|k|\cos\theta_{n}+e_{x}\left|k\right|\sin\theta_{n}\\ k_{n}^{R} & =-e_{y}|k|\cos\theta_{n}+e_{x}\left|k\right|\sin\theta_{n}\\ k_{\mathrm{inc}} & =e_{y}\left|k\right| \end{array}$$

with $\left|k\right|=2\pi f/c_{0}$, *θ*_*n*_ being the refraction angle, and **e** being the basis vectors. The transmission and the reflection coefficients then follow as

(6) $$\begin{array}{cc} & T_{n}=\frac{a_{n}^{T}}{a_{\mathrm{inc}}}\\ & R_{n}=\frac{a_{n}^{R}}{a_{\mathrm{inc}}} \end{array}$$

and the absorption coefficient as

(7) $$\alpha =1-\sum_{n}|T_{n}{|}^{2}-\sum_{n}{\left|R_{n}\right|}^{2},\;n\in \left\{0,\pm 1\right\}$$

The transmitted energy distribution follows as (Equation (3))

(8) $$\tau_{n}^{\mathrm{FEM}}=\frac{|T_{n}{|}^{2}}{|T_{-1}{|}^{2}+|T_{0}{|}^{2}+{\left|T_{+1}\right|}^{2}}$$

A 2D FEM model with finite metagrating including the adjacent geometries was used for calculating the diffraction pattern in Figure 3c,f,i. Only the domain around the metagrating was modeled including thermoviscous effects, while the remaining domains were modeled using the lossless Helmholtz equation, see Supporting Information for corresponding sound fields. Due to the large aperture and the resulting large number of elements, especially in the thermoviscous domain, a 3D simulation was not practical, therefore a 2D model was used. In addition, the precise geometry of the region around the sound source and the material parameters are uncertain, and we believe this explains the discrepancies in the 0th diffraction order in Figure 3c.

The material parameters used for air are equilibrium density *ρ*_0_ = 1.2 kg m^−3^, speed of sound *c*_0_ = 343 m s^−1^, dynamic viscosity μ = 1.814 × 10^−5^ Pa s, bulk viscosity μ_B_ = 1.0884 × 10^−5^ Pa s, thermal conductivity *k* = 0.025768 W m^−1^ K^−1^, heat capacity at constant pressure *C*_*p*_ = 1005.4 J kg^−1^ K^−1^, and ratio of specific heats *γ* = 1.4

Shape optimization was used to deform an existing meta‐atom geometry to improve its performance. The shape optimization of designs B and C used the Free Shape Boundary method. In this case, the boundary of the geometry to be deformed was moved while the FEM mesh was deformed around the geometry according to the smoothing type. Here, the three‐term Yeoh hyperelastic model, which was a generalization of a neo‐Hookean material, was applied with a stiffening factor of ten.^[^ ^45^ ^]^ The final geometries of designs B and C were obtained after 26 and 101 iterations, respectively. To create the initial state for the shape optimization of design C, a topology optimization using the density model^[^ ^51^ ^]^ in combination with the Helmholtz equation was applied. In this case, the mesh remains unchanged and a domain control variable was changed as it was discretized on the mesh. The filter type was set to Helmholtz,^[^ ^52^ ^]^ the projection type to hyperbolic tangent, the interpolation type to linear, and the element order to linear.^[^ ^45^ ^]^ The intermediate geometry of design C was obtained after 48 iterations.

### Sample Manufacturing Using Two‐Photon Lithography

The fabrication of samples was conducted using two‐photon polymerization based additive manufacturing. Processing was carried out using the Photonic Professional GT2 (Nanoscribe GmbH, Germany) with a build volume of 10 × 10 × 0.8 cm^3^.^[^ ^53^ ^]^ The voxel shape was elliptic, its size (0.1 µm < *r*_*v*_ < 5 µm and 0.3 µm < *z*_*v*_ < 15 µm^[^ ^53^ ^]^) depended on the illumination process parameters and the optical properties of the photoresist. The required structures could be created by scanning the voxel through the photoresist following positional data from CAD models. Excess photoresist was removed leaving the cured structures, which were detached from the substrate after drying.

Focusing was conducted by means of immersion objectives 10 × numerical aperture (NA) 0.3 and 25 × NA 0.8 for the grating holder and grating sample, respectively. The photoresists IP‐Q and IP‐S were used, respectively (Nanoscribe GmbH, Germany). The development of the photoresist was performed by 1‐methoxy‐2‐propylacetat (20 min, 60 mL) and 2‐propanol (5 min, 60 mL). Drying was carried out in air at 21.7 ± 0.44 °C and 32.1±8.2% relative humidity under a glass hood. The resulting mechanical properties for IP‐S were Poisson's ratio *ν*_IP‐S_ ≈ 0.3, Young's modulus *E*_IP‐S_ ≈ 5.11 GPa, and density *ρ*_IP‐S_ ≈ 1.22 g  cm^−3^.^[^ ^53^ ^]^

### Experimental Setup

As shown in Figure 2e, the measurement setup consisted of an assembly that connects a sound source with a microacoustic metagrating. In addition, the assembly was mounted in a position system (not shown) that enables the assembly to move relative to a microphone. The sound source was an in‐house designed and fabricated capacitive micro‐machined ultrasound transducer,^[^ ^54^ ^]^ which was mounted on TO‐18 (transistor‐style metal case, see Figure 2e, brassy) and clamped into the frame (see Figure 2e, pink) using the screw (see Figure 2e, green). In order to measure ultra‐high frequency ultrasound, the optical microphone Eta450 Ultra (Xarion, Austria) with a specified frequency range from 50kHz to 2 MHz was used.^[^ ^55^, ^56^ ^]^ The frequency range in this work could be extended to 2.2 MHz since only relative sound pressure values were used.

To measure radial scans in one plane motorized stages (Physical Instruments, Germany) were used. Since the microphone was aligned with the same side to the axis of rotation on the metagrating, the directional characteristic of the microphone were neglected.^[^ ^57^ ^]^ The measured angle lies within −70° < *θ* < +70° due to prevent collision between the rotatable holder and the microphone attachment. To reduce the measurement time, the radial axis was constantly moved and triggers sound pressure measurements at the chosen resolution of 0.1°. In order to accurately adjust the tilt and the positioning of the assembly including the source and the grating relative to the coordinate system of the motorized stage an adjustment laser was used. The same adjustment laser was used to ensure that the metagrating is in the center of the rotation. To determine the distance between transducer and the microphone a time of flight measurement with an pulsed stimulation and without metagrating was performed. The measurements were done with continuous stimulation (sinusoidal signal 10 Vpp + DC bias 20 V). The metagrating was aligned with the rotation axis, which was orthogonal to the measurement plane, while center of the microphone was located at the half of the height of the metagrating. The refracted wave was then measured using the optical microphone on a circular trajectory on a radius of 10.66 mm (design A), 8.22 mm (design B), and 8.19 mm (design C) from the metagrating surface.

### Experimental Data Analysis

The raw measured pressure amplitude was fitted using a Gaussian mixture model of the form

(9) $$\begin{array}{cc} & G\left(\theta \right)=\sum_{n}{\hat{p}}_{n}g_{n}\left(\theta \right)\\ & n\in \left\{0,\pm 1\right\}, \end{array}$$

with

(10) $$g_{n}\left(\theta \right)=e^{-\frac{{\left(\theta -\theta_{n}\right)}^{2}}{2\sigma^{2}}}$$

where ${\hat{p}}_{n}$ is the pressure amplitude at the beam center and σ corresponds to the beam width. The pressure magnitude error is estimated according to

(11) $$\Delta {\hat{p}}_{n}=\sqrt{\int_{\Theta }{\left(p_{exp}\left(\theta \right)-{\hat{p}}_{n}g_{n}\left(\theta \right)\right)}^{2}w_{n}\left(\theta \right)d\Theta }$$

with the weighting function *w*_*n*_(*θ*) = *g*_*n*_(*θ*). The pressure amplitude ${\hat{p}}_{n}$ was then used to determine the normalized transmission within the diffraction orders

(12) $$\tau_{n}^{\exp}=\frac{{\hat{p}}_{n}^{2}}{\sum_{m}{\hat{p}}_{m}^{2}},\;m\in \left\{0,\pm 1\right\}$$

which is linked to Equation (3). This quantity was introduced because of the difficulty of measuring absolute power and taking multiple reflections into account. Subsequently, the propagation of the error is determined as

(13) $$\Delta \tau_{i}^{\exp}=2\sqrt{\frac{{\hat{p}}_{i}^{4}\left(\Delta {\hat{p}}_{j}^{2}{\hat{p}}_{j}^{2}+\Delta {\hat{p}}_{k}^{2}{\hat{p}}_{k}^{2}\right)+\Delta {\hat{p}}_{i}^{2}{\hat{p}}_{i}^{2}{\left({\hat{p}}_{j}^{2}+{\hat{p}}_{k}^{2}\right)}^{2}}{{\left({\hat{p}}_{i}^{2}+{\hat{p}}_{j}^{2}+{\hat{p}}_{k}^{2}\right)}^{4}}}$$

with *i* ∈ { − 1, 0, +1}, *j* ∈ {0, +1, −1}, and *k* ∈ { + 1, −1, 0}, leading to the errorbars in Figure 3b,e,h.

## Conflict of Interest

The authors declare no conflict of interest.

## Author Contributions

A.M. designed and planned the study. A.M., Y.K.C., and D.P. conceptualized the general grating parameters. A.M., S.K., and S.S. conceptualized and designed the experimental setup. Y.K.C. implemented the semianalytical optimization method and created design A. A.M. implemented the optimization with thermoviscous losses and created designs B and C. S.K. built the experimental setup and performed the measurements. S.S. created 3D‐CAD models and manufactured the samples using two‐photon lithography. S.K. and S.S. took the photographs and the micrographs. A.M. analyzed the experimental data and prepared the figures. S.M. and D.P. supervised the work. All authors contributed to editing the draft of the manuscript.

## Supporting information

Supporting Information

## Data Availability

The data that support the findings of this study are available from the corresponding author upon reasonable request.
