# Supplementary material for: Microacoustic Metagratings at Ultra‐High Frequencies Fabricated by Two‐Photon Lithography
Source: Adv Sci (Weinh). 2022 Apr 24;9(20):2200990. doi: 10.1002/advs.202200990 (PMC9284164; doi:10.1002/advs.202200990)
Supplement: Supplementary file 1 — Supporting Information [file ADVS-9-2200990-s001.pdf]

Microacoustic metagratings at ultra-high frequencies fabricated by two-photon lithography
Supplementary Material

Anton Melnikov,[1,*] Sören Köble,[1] Severin Schweiger,[1] Yan Kei Chiang,[2] Steffen Marburg,[3] and David A. Powell[2]

[1] *Fraunhofer Institute for Photonic Microsystems, Dresden, Germany*

[2] *School of Engineering and Information Technology University of New South Wales, Canberra, Australia*

[3] *Chair of Vibro-Acoustics of Vehicles and Machines Technical University of Munich, Germany*

[*] anton.melnikov@ipms.fraunhofer.de

Figure S1 shows numerical results including thermovicous effects for the designs A, B, and C. We observe for design A in Fig. S1a that the transmitted pressure field at the target frequency 2 MHz is though dominated by −1st diffraction order, but shows a significant contribution of other orders. In Fig. S1b the absolute transmission to the −1st order is only $|T_{-1}|^2 \approx 0.2$ and the absorption is above $\alpha = 0.5$. Besides the absorption, the energy leaks into 0th transmitted and reflected diffraction orders. The situation is changed when considering the design B, which geometry is shown in Fig. S1c. The pressure field in Fig. S1c shows deviation of pressure distribution between the metaatoms and demonstrates a higher amplitude of the −1st transmitted diffraction order. Figure S1d shows a significant improvement considering −1st diffraction order with $|T|^2 \approx 0.45$ around the target frequency. Furthermore, the absorption and the unwanted diffraction orders are reduced compared to Fig. S1b. The shape of design C is shown in Fig. S1e, where we note that the thickness is much smaller compared to the designs A and B. Furthermore, the transmitted pressure field in Fig. S1e is strongly dominated by the −1st diffraction order. Even if the absolute transmission in Fig. S1f with $|T|^2 \approx 0.40$ at the target frequency is slightly reduced compared to design B in Fig. S1d, this design demonstrates a better broadband transmission being less sensitive to the frequency shifts and manufacturing tolerances. Figure S2 shows the resulting sound field generated by a finite microacoustic metagrating in combination with a sound source and considering neighboring geometries.

Figure S3 shows the electrical impedance of the used capacitive micromachined ultrasonic transducer. The center frequency of the transducer is exactly at the target frequency of 2 MHz and is indicated by the peak of the real part of the impedance R. This peak corresponds to the mechanical resonance of the transducer membrane, where depends on the bias voltage U_{DC}. We note, that the transducer demonstrates strong mechanical response within the whole range from 1.8 MHz to 2.2 MHz being measured experimentally.

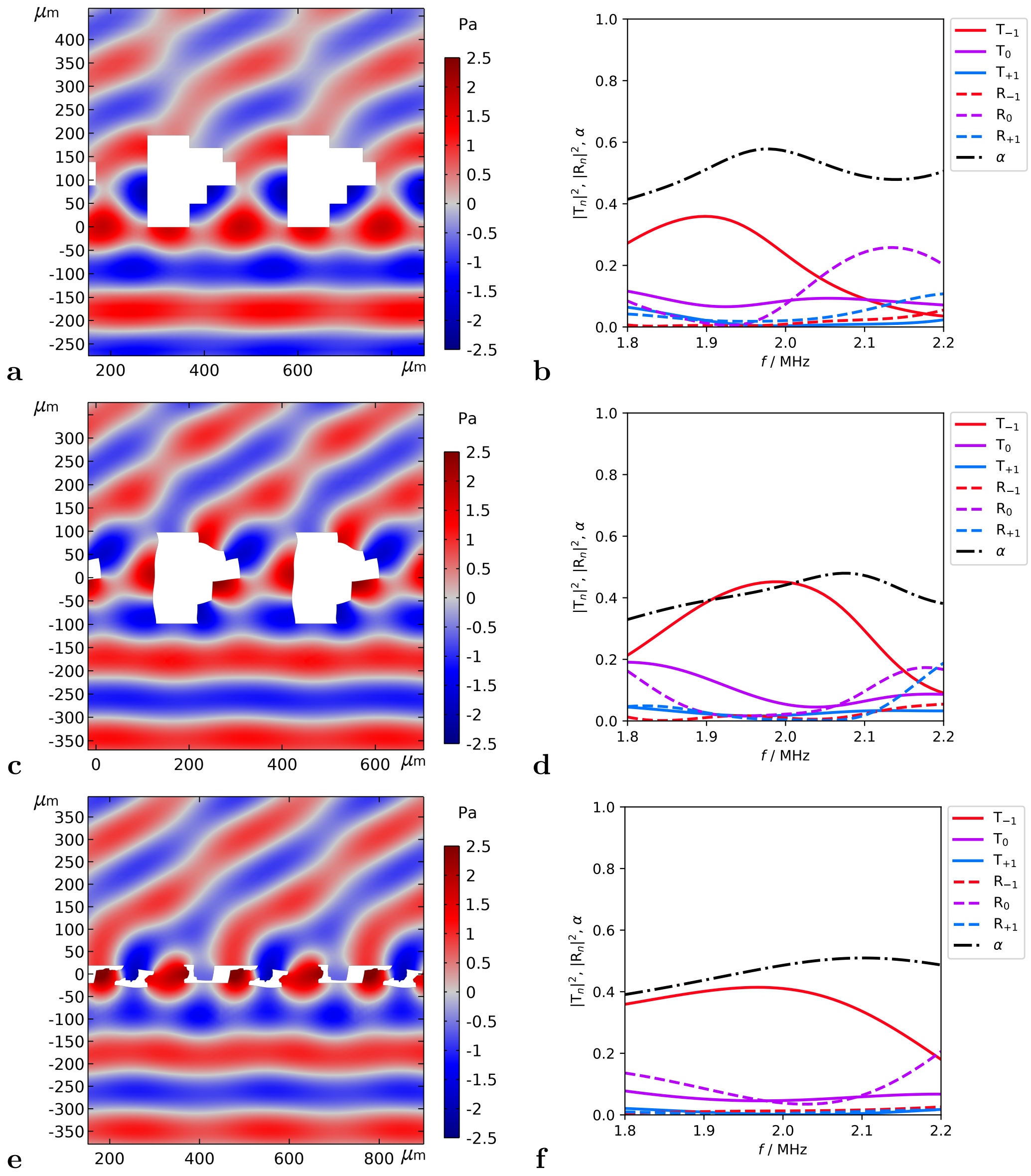

FIG. S1. **Numerical results for infinite microacoustic metagratings.** **ab** Design A, **cd** Design B, and **ef** Design C. **ace** Geometric shape of the metaatom and the real part of the pressure field at target frequency. **bdf** Unnormalized tranmission and reflection coefficients for infinite structure.

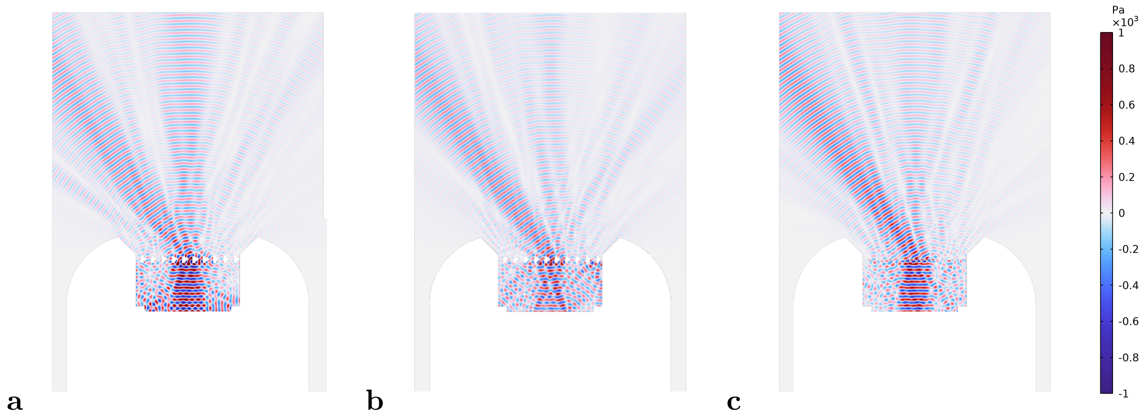

FIG. S2. **Sound field for finite microacoustic metagratings.** **a** Design A at 2.00 MHz, **b** Design B at 2.02 MHz, and **c** Design C at 2.00 MHz.

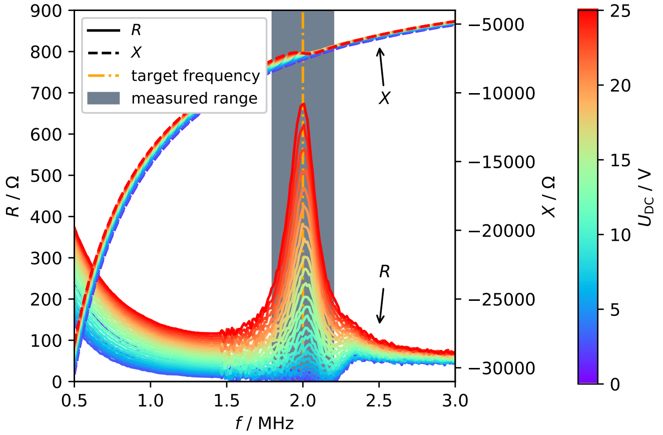

FIG. S3. Electrical impedance of the used transducer showing strong mechanical response in the measurement range from 1.8 MHz to 2.2 MHz (gray area), while the center frequency 2 MHz matches exactly the target frequency in that study (orange dash-dotted line). The impedance depends on the bias voltage U_{DC}, with different colors (see colorbar) corresponding to different bias voltages. The mechanical resonance is mainly observed in the real part X (solid lines) and less in the imaginary part R (dashed lines) of the impedance.
